# Fauna and Larval Habitats Characteristics of Mosquitoes (Diptera: Culicidae) in Golestan Province, Northeast of Iran, 2014–2015

**Published:** 2018-09-30

**Authors:** Aioub Sofizadeh, Hamid Reza Shoraka, Fatemeh Mesgarian, Ghorban Mohammad Ozbaki, Abdolsamad Gharaninia, Ebrahim Sahneh, Rohollah Dankoob, Ali Malaka, Saeid Fallah, Shamsaddin Nemani

**Affiliations:** 1 Infectious Diseases Research Center, Golestan University of Medical Sciences, Gorgan, Iran; 2 North Khorasan University of Medical Sciences, Bojnurd, Iran; 3 Health Centers of Health Deputy, Golestan University of Medical Sciences, Gorgan, Iran

**Keywords:** Culicidae, Larval habitat, Ecology, Iran

## Abstract

**Background:**

Mosquitoes (Diptera: Culicidae) is one of the most medically important families of Diptera. The aims of this study were to determine fauna and larval habitat characteristics of mosquitoes in Golestan Province, during 2014–15.

**Methods:**

This study was conducted in larval habitats of mosquitoes and installed ovitraps in 14 districts of Golestan Province, Northern Iran in 2015. Samples were collected with a scoop by ladle handling for entomology. The collected larvae were transferred to Laboratory of Medical Entomology in lactophenol solution. Then microscopic slides were prepared using de Faure’s formula. Species of each sample was recognized using diagnostic criteria to identify the Culicidae species. Characteristics of larval breeding places were studied based on the habitat type (Permanent or temporary), water conditions (Clear or turbid, stagnant or running), vegetation (out, in, underwater vegetation or without vegetation), sunlight exposure (Full or partial sunlight) and so on. Data were analyzed using SPSS.

**Results:**

Overall, 5661 third- and fourth- instars larvae of mosquitoes were collected and 5 genera and 14 species were identified: *Anopheles hyrcanus*, *An. maculipennis*, *An. pseudopictus*, *An. superpictus*, *Culex hortensis*, *Cx. mimiticus*, *Cx. perexiguus*, *Cx. pipiens*, *Cx. pusillus*, *Cx. theileri*, *Cx. tritaeniohynchus*, *Culiseta longiareolata*, *Ochlerotatus caspius*, *Uranotaenia unguiculata. Culex pipiens* was recognized as predominant species of the family. Among the detected species, *Cx. pusillus* reported for the first time from Golestan Province.

**Conclusion:**

Due to the high species diversity of Culicidae, ecology of medical important species such as *Cx. pipiens* and *Cx. tritaeniorhynchus* needs more investigations.

## Introduction

Mosquitoes (Diptera: Culicidae) as one of the most important families of insects. According to the latest taxonomy, Culicidae comprises two subfamilies, 11 tribes, 112 genera and 3539 species (1). The checklist of the mosquitoes in Iran includes 7 genera, 16 subgenera, 64 species and three subspecies (2).

Subsequently, *Anopheles superpictus* is two species in Iran based on the Internal Transcribed Spacer 2 (ITS2) sequences of rDNA (3), later listed as species A and B (4). A new species of the *Anopheles hyrcanus* group (*An. hyrcanus* spIR) was recognized from southwestern Iran also based on ITS2 sequence data (5). More recently, the occurrence of *Aedes albopictus* was reported in southeastern Iran and *Orthopodomyia pulcripalpis* in northern Iran, respectively (6, 7).

The fauna of mosquitoes of Golestan Province, Northern Iran includes *Aedes* (*Aedimorphus*) *vexans*, *Ae.* (*Dahliana*) *echinus*, *Ae.* (*Dah.*) *geniculatus*, *Ae.* (*Ochlerotatus*) *caspius*, *Ae.* (*Och*.) *pulcritarsis*, *Anopheles alrgeriensis*, *An. claviger*, *An. hyrcanus*, *Anopheles. maculipennis* s.l., *An. melanoon*, *An. multicolor*, *An. plumbeus*, *An. pseudopictus*, *An. pulcherrimus*, *An. superpictus*, *Coquillettidia richiardii*, *Culex hortensis*, *Cx. mimeticus*, *Cx. perexiguus*, *Culex. pipiens*, *Cx. territans*, *Cx. theileri*, *Culiseta longiareolata*, *Cs. subochrea*, *Uranotaenia unguiculata* (8–13). Mosquitoes are important vectors of many diseases including: malaria, West Nile virus, dengue fever, yellow fever, filariasis and other diseases (14). Some of these diseases and their agents have previously been reported from Golestan Province, for example: malaria, West Nile Virus, *Dirofilaria immitis* (15–17).

Malaria as one of the most important vector-borne diseases in Iran is transmitted by *Anopheles* mosquitoes. Over the past decades, north of the country including Golestan Province has been identified as one of the most important endemic foci of malaria (15), eight species of the genera *Anopheles* are known as vectors of malaria in Iran. *An. culicifacies* s.l., *An. dthali*, *An. fluviatilis* s.l., *An. maculipennis* s.l., *An. sacharovi*, *An. stephensi*, and *An. superpictus* have been introduced as primary and secondary malaria vectors and *An. pulcherrimus* as a suspected vector (11). West Nile virus is a mosquito-borne virus that transmitted by *Culex* species to birds, equines, and humans (18) seropositive cases of humans and equines for these viruses were reported in Golestan Province (16).

*Dirofilaria immitis* also is one of the most important mosquito-borne pathogens that reported in Golestan Province (17), *Culex theileri* is known vector of this pathogen in the north-west of Iran (19).

Mosquitoes play a key role in transmitting diseases, many have studies carried out in north of Iran and Golestan Province on the biology and ecology of mosquitoes: larval habitats characteristics of mosquitoes and biological characteristics of *Anopheles* was reported in Kalaleh County, Golestan Province (12, 13). Fauna and larval habitats of mosquitoes were reported in Guilan and North Khorasan Provinces, north of Iran (20–23). Fauna, larval habitat and other biological characteristics of mosquitoes were reported in Mazandaran Province and Neka County (24–26). Physicochemical characteristics of mosquitoes were studied in Qom Province (27) and larval habitats, affinity and diversity indices of Culicinae in Southern Iran (28). Different species of mosquitoes require different biological and ecological conditions to growth and development. While some lay eggs and breed in permanent water habitats, others prefer temporary water bodies for breeding, some favour feeding on sweet water, others need salty water, and some prefer high temperature and humidity while others are in favour of low temperature and humidity. Regardless of these differences, is clear and evident that all need water for breeding and without water, their chances for growth and development would be slight.

Though the climate in Golestan Province is suitable for the growth and development of mosquitoes such as *Anopheles*, *Aedes* and *Culex* mosquitoes, so far, no comprehensive studies have been carried out on the fauna and ecological properties of mosquitoes in Golestan Province. To address this gap, this research was an attempt to analyze the characteristics of mosquito larval habitats.

## Materials and Methods

### Study area

**Fig. 1.**
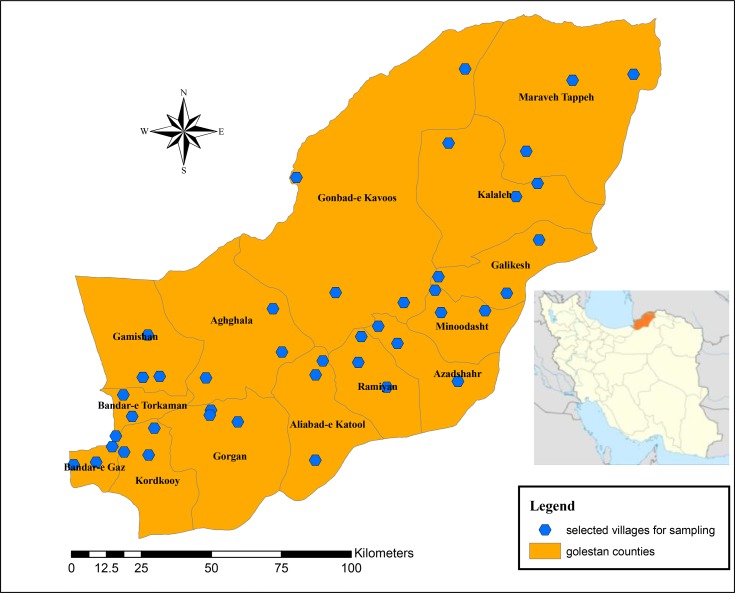
Location of Golestan Province and selected villages for sampling of Culicidae

Golestan Province (53°57′–56°23′ E, 36° 30′–38°08′ N) covering a landmass of 20437.74 square km consists of approximately 1.3% of the total area of Iran. It is located in the northeastern region of the country and bordered by the Republic of Turkmenistan to the north, Alborz Mountain range and Semnan Province to the south, North Khorasan Province to the east, and Caspian Sea and Mazandaran Province to the west. This province consists of 14 counties, 25 cities, 60 districts and 1764 villages (Fig. 1). The province is enriched with diverse ecology and climatic conditions. Considering the sea, forest and mountainous areas, the climatic condition of the province is classified into temperate mountainous, cold mountainous (3000m high), a mild Mediterranean and arid and semi-arid regions, such that as we move from southern to northern parts, the amount of rainfall and relative humidity decreases and the degree of temperature increases. With regards to the topography of the province, this region is subdivided into three distinct areas; mountainous, plain and even posts. The mountainous areas are located in the southern parts containing the highest peaks of the province. Mountainous areas are located in the foothills of the southern and eastern borders of the province with coarse sediments as alluvial fans make use of this land. As a result of the high permeability of the soil in the mountainous areas, groundwater aquifers with water in wells and canals are exploited. The retreat of the Caspian Sea post and plain regions has created severe water erosion and compaction of alluvial rivers. The lowest parts of the province (around the Caspian Sea with an altitude of 32m above sea level) are located in an area inhabited by a majority of the province’s population (29).

### Specimen and data collection

This study was performed in all counties of Golestan Province, northern Iran in 2015. In each county, one city and two villages were selected based on the topographic conditions. In order to sampling of larves, in each selected city and villages, at least two larval habitats were searched. First, features such as larval habitat status (permanent or temporary, stagnant or slow-running water), vegetation type, substrate type, habitat types and position of the sunlight were recorded on special forms. Then, larval sampling method was carried out using standard dipper of 350ml. Each habitat was sampled in different parts of the larval habitats for 10 times. All captured larvae in each dipper were counted and collected in special containers. The information of habitats was recorded on the larva containers and transferred to the medical entomology laboratory in Health Center of Kalaleh County. In the laboratory, after drying, larvae were kept in lactophenol medium and were mounted on microscope slides in de Faure’s formula and using standard taxonomic keys (30), larvae species were determined. Sampling was done once in each of the seasons of spring, summer, and autumn.

### Preparation of Ovitrap and larva sampling of this trap for searching *Aedes* eggs and larva

We used CDC Ovitrap. This consists of a three-liter, black, water-filled, plastic container and thin paddle of wood (2×12.5cm) placed in the container (31, 32). In each selected city and village, 10 ovitraps were implemented and investigated once a week and collected the present larvae and as it was explained previously, they were mounted and Species of each sample was recognized.

## Results

Overall, 5661 larvae belonged to Culicidae were collected and their species were identified. Including *An. hyrcanus*, *An. maculipennis* s.l., *An. pseudopictus*, *An. superpictus* s.l., *Cx. hortensis*, *Cx. mimeticus*, *Cx. perexiguus*, *Cx. pipiens*, *Cx. pusillus*, *Cx. theileri*, *Cx. tritaeniohynchus*, *Cs. longiareolata*, *Oc. caspius*, and *Uranotaenia unguiculata.*

Of 2821 ones had been collected from natural larval habitats and 2840 ones from the prepared ovitraps (Table 1). *Culex pipiens* was identified as the dominant species of Golestan Province and it was collected from all counties of this province. In terms of frequency, *Cx. tritaeniohynchus* was in the second rank and was collected from a majority of counties (Table 2). In the present study, 77.3% of larvae were collected from temporary larval habitats compared to permanent ones and 73.6% of larvae from larval habitats with stagnant water compared to those with running water (Table 3). More larvae were collected from natural larval habitats of wetlands (31.3%) and artificial larval habitats of bogs (26.4%) (Table 4, 5).

**Table 1.** Abundance of mosquito (Culicidae) larvae in Golestan Province, Nortehrn Iran 2015

**species**	**Natural larval habitats**		**Ovitrap**		**Total**	

	**Num.**	**%**	**Num.**	**%**	**Num.**	**%**
***An. hyrcanus***	2	0.1	0	0	2	0.001
***An. maculipennis***	20	0.7	0	0	20	0.4
***An. pseudopictus***	14	0.5	0	0	14	0.2
***An. superpictus***	178	6.3	0	0	178	3.1
***Cx. hortensis***	6	0.2	0	0	6	0.1
***Cx. mimeticus***	46	1.6	2	0.1	48	0.8
***Cx. perexiguus***	72	2.5	7	0.2	79	1.4
***Cx. pipiens***	1657	58.2	2736	96.3	4393	77.3
***Cx. pusillus***	1	0.001	3	0.1	4	0.1
***Cx. Theileri***	5	0.2	4	0.1	9	0.2
***Cx. tritaeniohynchus***	617	21.7	88	3.1	705	12.4
***Cs. longiareolata***	18	0.6	0	0	18	0.3
***Oc. caspius***	183	6.4	0	0	183	3.2
***Uranotaenia unguiculata***	2	0.1	0	0	2	0.001
**Total**	**2821**	**100**	**2840**	**100**	**5661**	**100**

**Table 2.** Distribution of mosquitoes in different counties of Golestan Province, northern Iran

**Counties**	**Maraveh Tapeh**	**Kalaleh**	**Gonbad-e Kavus**	**Galikesh**	**Minoodasht**	**Azadshahr**	**Ramian**	**Aliabad-e Katul**	**Aq Qala**	**Gorgan**	**Kordkouy**	**Bandar-e Gaz**	**Bandar-e Turkman**	**Gomishan**	**Total**
**Species**															
***An. hyrcanus***	0	0	0	0	2	0	0	0	0	0	0	0	0	0	2
***An. maculipennis***	0	15	0	5	0	0	0	0	0	0	0	0	0	0	20
***An. pseudopictus***	0	0	0	0	14	0	0	0	0	0	0	0	0	0	14
***An. superpictus***	0	178	0	0	0	0	0	0	0	0	0	0	0	0	178
***Cx. hortensis***	0	0	0	6	0	0	0	0	0	0	0	0	0	0	6
***Cx. mimeticus***	0	5	0	18	24	0	0	1	0	0	0	0	0	0	48
***Cx. perexiguus***	63	1	0	8	3	0	0	0	0	0	0	3	1	0	79
***Cx. pipiens***	164	152	1658	60	156	223	161	564	40	80	742	265	74	54	4393
***Cx. pusillus***	3	1	0	0	0	0	0	0	0	0	0	0	0	0	4
***Cx. theileri***	4	0	3	0	0	0	0	0	0	0	0	2	0	0	9
***Cx. tritaeniohynchus***	53	62	221	103	59	0	5	49	4	0	5	59	85	0	705
***Cs. longiareolata***	0	18	0	0	0	0	0	0	0	0	0	0	0	0	18
***Oc. caspius***	0	0	104	0	0	0	0	28	0	0	0	0	51	0	183
***Uranotaenia unguiculata***	0	1	0	0	0	0	0	0	0	0	0	0	1	0	2
**Total**	**287**	**433**	**1986**	**200**	**258**	**223**	**166**	**642**	**44**	**104**	**747**	**329**	**212**	**54**	**5661**

**Table 3.** Larval habitat characteristics of mosquitoes collected in Golestan Province, northern Iran 2015

**Species**	***An. hyrcanus***	***An. maculipennis***	***An. pseudopictus***	***An. superpictus***	***Cx. hortensis***	***Cx. mimeticus***	***Cx. perexiguus***	***Cx. pipiens***	***Cx pusillus.***	***Cx. theileri***	***Cx. tritaeniohynchus***	***Cs. longiareolata***	***Oc. caspius***	***Uranotaenia unguiculata***	**Total**
**Larval habitats characteristics**															
**Habitat situation**															
**Permanent**	2	0	7	29	4	2	33	488	1	0	56	0	25	0	645
**Temporary**	0	20	7	148	4	44	39	1169	0	5	561	18	158	20	2193
**running water**	2	0	7	83	4	2	8	523	1	2	118	0	0	0	750
**Stagnant water**	0	20	7	95	2	44	64	1134	0	3	499	18	183	20	2089
**Vegetation situation**															
**Out of water**	0	0	0	121	0	34	56	527	0	5	282	0	104	0	1165
**In water level**	0	0	0	0	4	12	6	370	0	0	136	0	28	0	571
**underwater**	0	0	0	0	0	0	8	49	0	0	2	0	26	0	85
**without**	0	0	0	57	2	0	2	711	1	0	197	18	25	0	1013
**Sunlight situation**															
**Full sunlight**	0	0	0	28	0	5	30	537	0	5	389	0	127	0	1124
**Shaded**	0	0	0	0	0	0	0	64	0	0	20	0	30	0	118
**Partial sunlight**	2	5	7	26	0	19	38	399	0	0	101	0	10	0	607
**Sunlight shaded**	0	15	7	124	0	22	4	657	1	0	110	18	16	1	975
**Substrate**															
**mud**	2	19	14	149	6	46	72	1004	0	5	481	0	158	2	1958
**sand**	0	1	0	29	0	0	0	279	1	0	136	0	25	0	471
**rock**	0	0	0	0	0	0	0	70	0	0	0	0	0	0	70
**others**	0	0	0	0	0	0	0	295	0	0	0	18	0	0	313
**Water Situation**															
**muddy**	0	0	0	28	0	0	5	372	0	0	48	0	0	0	453
**clear**	2	20	14	150	6	46	66	1114	1	4	497	18	155	1	2094
**Turbid**	0	0	0	0	0	0	1	171	0	1	72	0	28	1	274
**fresh**	2	20	14	95	6	46	29	1571	0	5	494	18	158	1	2459
**salty**	0	0	0	0	0	0	26	34	0	0	18	0	0	0	78
**brackish**	0	0	0	83	0	0	17	52	1	0	105	0	25	1	284
**Habitat Kind**															
**Natural**	2	5	14	83	6	41	28	446	1	4	310	2	183	1	1126
**Artificial**	0	15	0	95	0	5	44	1211	0	1	307	16	0	1	1695

**Table 4.** Abundance of species of mosquitoes in natural habitats in Golestan Province, northern Iran, 2015

**Natural habitats**	**River Edge**	**Riverbed**	**Marsh**	**Creek**	**Fountain**	**Pit**	**Wetlands**	**Tree holes**	**Water leakage**	**Total**
**Type Species**										
***An. hyrcanus***	2	0	0	0	0	0	0	0	0	2
***An. maculipennis***	0	0	0	0	0	0	5	0	0	5
***An. pseudopictus***	7	0	7	0	0	0	0	0	0	14
***An. superpictus***	29	0	0	26	0	0	28	0	0	83
***Cx. hortensis***	0	2	0	4	0	0	0	0	0	6
***Cx. mimeticus***	2	0	10	0	0	7	5	0	17	41
***Cx. perexiguus***	1	0	0	8	6	2	8	0	3	28
***Cx. pipiens***	9	9	13	52	0	109	144	0	37	446
***Cx. pusillus***	1	0	0	0	0	0	0	0	0	1
***Cx. theileri***	0	0	0	2	0	2	0	0	0	4
***Cx. tritaeniohynchus***	73	2	16	9	2	10	124	0	74	310
***Cs. Longiareolata***	0	0	0	0	0	0	0	2	0	2
***Oc. caspius***	0	0	25	104	0	16	38	0	0	183
***Uranotaenia unguiculata***	0	0	0	0	0	0	1	0	0	1
**Total**	197	13	71	205	8	146	353	2	131	1126

**Table 5.** Abundance of species of Culicidae in artificial habitats in Golestan Province, northern Iran, 2015

**Artificial habitats type**	**Create sides of rice**	**Create middle of rice**	**Stream of creating rice**	**Bog**	**Other agricultural streams**	**Cistern**	**Lake**	**Well**	**Pool**	**Others**	**Total**
**species**											
***An. hyrcanus***	0	0	0	0	0	0	0	0	0	0	0
***An. maculipennis***	15	0	0	0	0	0	0	0	0	0	15
***An. Pseudopictus***	0	0	0	0	0	0	0	0	0	0	0
***An. superpictus***	95	0	0	0	0	0	0	0	0	0	95
***Cx. hortensis***	0	0	0	0	0	0	0	0	0	0	0
***Cx. mimeticus***	0	0	0	0	0	0	0	5	0	0	5
***Cx. perexiguus***	44	0	0	0	0	0	0	0	0	0	44
***Cx. pipiens***	114	25	57	446	153	0	48	0	94	274	1211
***Cx. pusillus***	0	0	0	0	0	0	0	0	0	0	0
***Cx. theileri***	1	0	0	0	0	0	0	0	0	0	1
***Cx. tritaeniohynchus***	33	12	48	1	199	0	0	9	0	5	307
***Cs. longiareolata***	0	0	0	0	0	16	0	0	0	0	16
***Oc. caspius***	0	0	0	0	0	0	0	0	0	0	0
***Uranotaenia unguiculata***	0	0	0	0	0	0	0	0	0	1	1
**Total**	302	37	105	447	352	16	48	14	94	280	1695

## Discussion

A total of 5661 larvae belonged to 5 genera and 14 species were identified: *Anopheles* genus: 4 species, *Culex* genus: 7 species, *Culiseta*, *Ochlerotatus*, and *Uranotaenia* genera: one species. In previous studies of authors in Kalaleh County, *An. hyrcanus*, *An. maculipennis* s.l., *An. pseudopictus*, *An. superpictus*, *Cx. hortensis*, *Cx. perexiguus, Cx. pipiens*, *Cx. theileri*, *Cs. Longiareolata* and *Oc. caspius* had been reported (12, 13). Furthermore, species such as *An. claviger* and *Cs. subochrea* identified in Kalaleh study (13) were not collected in the present study.

In previous studies of authors in Kalaleh County, *Cx. pipiens*, *Cx. theileri*, *Cx. hortensis*, *Cx. perexiguus*, *An. maculipennis* s.l., *An. superpictus*, *An. hyrcanus*, *An. pseudopictus*, *Oc. caspius* and *Cs. longiareolata* had been reported (12, 13). Furthermore, species such as *An. claviger* and *Cs. subochrea* identified in Kalaleh study (13) were not collected in the present study. In some further studies in north of Iran and Golestan Province related to the fauna and ecology of mosquitoes (8–13) were identified species such as: *Aedes vexans*, *An. alrgeriensis*, *An. claviger*, *An. melanoon*, *An. multicolor*, *An. plumbeu*, *An. pulcherrimus*, *Coquillettidia richiardii*, *Cx. territans*, *Cs. subochrea*, *Oc. echinus*, *Oc. geniculatus*, *Oc. pulcritarsis* were not collected in our study, and *Cx. pusillus* were reported for the first time from Golestan Province.

In the present study, the dominant species was *Cx. pipiens* and 77.3% of the whole collected larvae belonged to this species. In the studies of Mazandaran Province and Kalaleh County, (13, 25, 26), Moreover, *Cx. pipiens* had been reported as dominant species. The dominant species had been *An. hyrchanus* in Neka County, Mazandaran Province (24) and *An. maculipennis* and *Cx. theileri* in Ardebil Province in 2008 (19). *Culex pipiens* has been reported from almost all provinces of Iran (33). In the present study, too, this species was reported from all counties of Golestan Province. Larva habitats of this species varied, but mostly of samples were collected from Bogs, agricultural streams, wetlands and rice fields. In Mazandaran, reported wetlands and discarded tires the main larval habitats for *Cx. pipiens* (26). In Isfahan (34), this species was mostly found in rice fields and natural habitats. Larval habitats of this species are mostly stagnant and artificial bodies of fresh water such as swamp of watering channels and holes and barrels filled with rain (35). Investigation of *Cx. pipiens* larval habitats and their characteristics showed that this species was collected from different larval habitats with various ecological conditions; even, 96% of the whole collected species from ovitraps belonged to this species and this species has a very high adaptability with various larval habitats and this factor has caused its high frequency and distribution reported from Iran and the region (33).

*Culex tritaeniohynchus* was one of the other species collected from a majority of counties and after *Cx. pipiens*, in the second rank, it included 12.4% of the whole collected larvae, 21.7% of larvae isolated from different larval habitats and also 3.1% of larvae isolated from ovitraps. Similar to our study, in Guilan, this species was in the second rank after *Cx. pipiens* (33), and in Mazandaran study (25), in the third rank after *Cx. pipiens* and *Cx. torrentium*, while in Neka County of Mazandaran Province, Kurdistan, Esfahan and Qom Provinces studies, no larvae from this species has been collected (15, 36–38). In Golestan Province, this species has been collected from different larval habitats, but its frequency in temporary and stagnant water habitats (95.7%) was more than that in permanent and running water habitats (4.3%). This observation is consistent with another study in Iran (21) but in Southern Iran (28), these species were collected in permanent water higher than temporary water habitats. Furthermore, the frequency of this species in full sunlight situation and Mud substrate habitats was higher than in Shaded situation and sandy substrates habitats. In southern Iran (28), these species were collected in full sunlight and sandy substrate habitats higher than partial sunlight and mud substrate habitats. This species was mostly found in agricultural streams, wetlands, and water leakage and river edge.

In this study, *Cx. perexiguus* was mostly found in Maraveh Tappeh County in the northeast of Golestan Province (80%), but it was found even in western counties of Golestan Province such as Bandar-e Gaz and Bandar-e Turkmen. These species prefers temporary larval habitats to permanent ones (13, 35, 39). In the present study, 55.5% of the collected larvae of this species were collected from temporary habitats. This species was only collected from natural habitats and no larvae were collected from artificial habitats, In Kalaleh study (13) 89% larvae of this species were collected from natural habitats and in central Iran study (37) larvae of *Cx. perexiguus* were only collected from rice fields as well in our study. The preferred habitats for this species were rice fields (61.1%). The notable point for habitats of this species is that it was collected from habitats with sweet, salty and brackish water. This feature was also true for only *Cx. pipiens* and *Cx. tritaeniohynchus* species. Since this species was collected from ovitraps and habitats with different conditions, it has good adaptability with different conditions of larva habitats.

From *Culiseta* genus, only *Cs. logiareolata* and from Kalaleh County was collected. The information about the ecology of *Culiseta* species in Iran is limited. In the present study, *Cs. logiareolata* was collected from only one larval habitat which was a destroyed cistern with cement substrate without vegetation. However, in Guilan Province, North Khorasan, Yazd and Esfahan provinces (20, 23, 34, 40) this species collected with high abundance and in other studies, this species had been collected from a variety of habitats (35, 36). In Yazd County, too, have reported *Cs. logiareolata* and *Cx. pipiens* in larval habitats infected with organic substances, industrial waste materials and cement pools for storing animals’ water (40).

*Anopheles maculipennis* Group, *An. claviger*, *An. hyrcanus*, *An. plumbeus*, *An. alrgeriensis* and *An. multicolor* and *An. pulcherrimus* have been previously reported in Golestan Province (8–12), but in the present study, *An. superpictus*, *An. maculipennis*, *An. psudopictus* and *An. hyrcanus* were collected and identified. *Anopheles superpictus* and *An. maculipennis* have been known as the vectors of malaria disease. In our study, *An. superpictus* was mostly found in the natural habitats of river beds and streams with permanent and stagnant water, also with or without vegetation. These findings are completely similar to the results reported in previous studies (13, 36, 40). In Guilan Province, this species only reported of natural habitats with transient, stagnant and clear water, and was mostly found in Rain pool habitats (23). The characteristics of larval habitats of *An. superpictus* are firstly shallow clear waters with stony bed and without vegetation and secondly rivers with sandy bed under sunlight and also shallow streams with muddy bed (34). Moreover, in the present study, this species was collected from both types of the mentioned larval habitats.

From genus *Ochlerotatus*, only *Oc. caspius* was collected, while in Kalaleh County study (13), three species of these genera had been reported (*Oc. caspius*, *Oc. echinus,* and *Oc. geniculatus*). *Ochlerotatus caspius* has been collected in many studies in Iran (13, 27, 41–43). In terms of frequency, in Kurdistan study (41), it was in the second rank after *Cx. theileri*. This high frequency of this species can be due to the *Ochlerotatus* mosquitos grow in humid forest regions and more selected villages for sampling are in forest regions. In relation to the characteristics of larval habitats, *Oc. caspius* preferred temporary, running water, muddy Substrate and Full sunlight habitats. In Kurdistan Province (36) this species reported of river edge with stagnant and clear water and partial sunlight habitat. Although this species prefers larval habitats with clear and sweet water, it was also collected in larval habitats with turbid and brackish water.

*Culex mimeticus*, *Cx. theileri*, *Cx. hortensis*, *Cx. pusillus*, *An. maculipennis* s.l., *An. hyrcanus*, *An. pseudopictus* and *Uranotaenia unguiculata* were collected with low frequency in Golestan Province; the larval habitat characteristics of these species must be extensively studied.

In addition, one of the aims of this study was to find *Aedes* genus larvae, but we did not collect any larvae of this genus in Golestan Province, although *Ae. vexans* was previously reported from the province (8).

## Conclusion

Due to the good climate conditions, different species of mosquitoes grow in Golestan Province and since there are different vectors for various diseases among these species, conditions of disease transfer are present in this region. In the present study, we did not collect any species among the vectors of diseases such as dengue fever, Zika, and chikungunya. Future studies for finding these vectors in Golestan Province are recommended.
